# The effect of group B streptococcus on maternal and infants’ prognosis in Guizhou, China

**DOI:** 10.1042/BSR20191575

**Published:** 2019-12-10

**Authors:** Wei Dai, Youcheng Zhang, Yin Xu, Mingjuan Zhu, Xiaotin Rong, Qing Zhong

**Affiliations:** Department of Obstetrics, Guizhou People’s Hospital, Guiyang 550002, Guizhou, China

**Keywords:** Group B Streptococcus, Late pregnancy, Neonatal outcome, Pregnancy outcome

## Abstract

Group B Streptococcus (GBS) is a kind of opportunistic pathogenic bacteria and mainly strikes the lower digestive tract and genitourinary tract. It is a major risk factor for neonatal babies, seriously threatening their lives. In the present study, we aimed to detect the GBS colonization in late pregnant women, and to study the effect of GBS on maternal and infants’ prognosis. Pregnant women with a gestational age of 35–37 weeks were enrolled in the study. Real-time polymerase chain-reaction (RT-PCR) was used to detect the colonization of GBS in the vaginal and rectal secretions for late pregnant women according to the screening guidelines. Chi-square test was applied to analyze the relationship between GBS colonization and clinical characteristics. A follow-up of 6 weeks was performed on the puerpera and infants after delivery. The positive rate of GBS was 12.6% in late pregnant women. GBS carrier state was positively related to several pregnancy outcomes, including intrauterine infection, premature rupture of membranes, postpartum hemorrhage, fetal distress and puerperal infection, as well as to part neonatal outcomes, containing neonatal infection, neonatal pneumonia and neonatal sepsis (all *P* < 0.05). GBS infection in late pregnant women results in adverse effects on maternal and neonatal outcomes.

## Introduction

Group B Streptococcus (GBS), also called as Streptococcus agalactiae, is a kind of gram positive bacteria and mainly lives in gastrointestinal tract, vagina and urethra [1]. GBS represents a major risk factor for infection among early neonatal babies, which always leads to their septicemia, pneumonia and meningitis, and seriously threatens their lives [2–8]. GBS infection in newborns shows diverse patterns within and between countries. Most women infected by GBS are asymptomatic, but the organism can be found in their throat, vagina and rectum. The prevalence of GBS infection in pregnant women is about 5–40% in different countries [9,10]. Early onset neonatal GBS infection mainly results from the aspiration of infected amniotic fluid or the birth canal transmission in childbirth [11–13]. In the presence of other predisposing factors like prematurity, maternal fever, premature rupture of membranes (PROM) more than 18 h, low birth weight and multiparity, the infection rate of GBS is increasing.

Reportedly, GBS is a normal flora in different organs, but during pregnancy, the risk of invasive GBS diseases is increased [14]. Meanwhile, GBS infection in pregnant women may lead to different adverse events for themselves. Furthermore, early onset GBS infection leads to severe neonatal condition, which may result in serious neurological damage. The latest U.S. Centers for Disease Control and Prevention (CDC) screening guidelines recommend pregnant women of 35–37 weeks to receive the screening of rectovaginal GBS [8]. The distribution of GBS varies across regions, studies exploring the effect of GBS on maternal and infants’ prognosis are necessary.

In the present study, our objective was to detect the GBS status of late pregnant women and to study the effect of GBS on maternal and infants’ prognosis.

## Methods and materials

### General information of pregnant women

A total of 380 pregnant women who had periodic antenatal inspection detected in Guizhou People’s Hospital were enrolled in the present study. Their pregnant weeks were confirmed and checked based on the end of menstruation and early pregnancy B ultrasound examination. Recruitment criteria for pregnant women contained the following aspects: (1) native population; (2) aged between 20 and 46 years old; (3) with a gestational duration between 35 and 37 weeks; (4) without the history of sexual intercourse or antibiotic application within the last 3 months. All samples and experiments were notified to pregnant women. Written informed consents were signed by both pregnant women and their families before participation. The present study was approved by the Ethics Committee of the hospital.

### TaqMan real-time polymerase chain-reaction (RT-PCR)

First, away the excess secretions of vulva was wiped away, and sterile cotton swab was entered into at 1/3 of the vagina and rotated a round to get vaginal secretions. Then, another sterile cotton swab was inserted into the anus, and collected rectal secretions 2–3 cm above the anal sphincter.

The swabs were placed in 2 ml stroke-physiological saline solution, transported and stored at room temperature, and tested within 24 h. Genomic DNA was extracted from the overnight cultures of GBS using Qiagen DNeasy Blood and Tissue kit according to the manufacturer’s instructions. Primers and probes referred to the previous study [15]. PCR reactions were performed on a StepOne Plus thermal cycler (Applied Biosystems) and analyzed using StepOne software. The test was performed in the molecular pathology laboratory of Department of Medicine in Guizhou People’s Hospital.

### Pregnancy/neonatal outcomes and follow-up

Maternal and fetal outcomes after childbirth were recorded, containing delivery mode, intrauterine infection, fetal infection, postpartum hemorrhage, fetal distress and other adverse events.

Intrauterine infection would be confirmed when any two of the following conditions were met: intrapartum maternal fever with a temperature ≥38°C; maternal pulse >100 times; fetal tachycardia with a heart rate >160 times continuously; pregnant women peripheral blood leukocyte count >150 × 10^9^/l, the proportion of neutrophils increased significantly; uterine tenderness; and amniotic fluid with bad smell.

Neonatal infection would be determined if puerperae had some high-risk factors, such as premature rupture of membranes and intrauterine infection; neonatal clinical manifestation appeared, like the temperature of neonatal did or did not rise, pale skin, listlessness and vomiting; and peripheral blood leukocyte count >25 × 10^9^/l.

All of the pregnant women were followed up for 6 weeks after delivery.

### Statistical analysis

All statistical analyses were performed using SPSS 19.0 statistical software. Chi-square test was applied to estimate difference in clinical characteristics between late pregnant women with different GBS infection statuses. Spearman test was used to analyze the clinical correlations of GBS with pregnancy/neonatal outcomes. *P* < 0.05 was considered statistically significant in the present study.

## Results

### General information of pregnant women

After PCR examination, 48 pregnant women were GBS positive, while 332 ones showed negative results. The average age was 30.04 ± 3.22 years among GBS positive women, and 30.67 ± 3.51 among negative ones, without significant difference between the two groups (*P* > 0.05). Besides, there were 312 primipara and 68 multipara. A total of 95 cases had a history of abortion, and 15 cases experienced multiple miscarriages (≥3 times).

### GBS distribution in pregnant women with different clinical factors

We also analyzed the distributions of GBS in pregnant women with different clinical factors and the results are shown in Table 1. Positive GBS rate was 11.63% (15/129) in elder pregnant women, and 21.05% (20/95) in those with abortion history. Moreover, positive GBS carriers were significantly more frequent in pregnant women with abortion history (*P* = 0.004), gestational diabetes mellitus (GDM) (*P* = 0.004) and pregnancy-induced hypertention (PIH) (*P* = 0.028) (Table 1).

**Table 1:** The clinical related factors of GBS carrier in late pregnant women

Factors	No.	GBS positive (*n* = 48)	GBS negative (*n* = 332)	χ^2^	*P*
Age				0.178	0.673
<35	251(%)	33(13.15)	218(86.85)		
≥35*	129(%)	15(11.63)	114(88.37)		
Abortion history				8.139	0.004
Yes	95(%)	20(21.05)	75(78.95)		
No	285(%)	28(9.82)	257(90.18)		
Gravida and parity				1.888	0.169
Primipara	312(%)	36(11.54)	276(88.46)		
Multipara	68(%)	12(17.65)	56(82.35)		
GDM				8.320	0.004
Yes	26(%)	8(30.77)	18(69.23)		
No	354(%)	40(11.30)	314(88.70)		
PIH				4.812	0.028
Yes	12(%)	4(33.33)	8(66.67)		
No	368(%)	44(11.96)	324(88.04)		
PO				1.174	0.279
Yes	3(%)	1(33.33)	2(66.67)		
No	377(%)	47(12.47)	330(87.53)		

* It was defined as advanced maternal age; GDM, gestational diabetes mellitus; PIH, pregnancy-induced hypertention; PO, pregnancy obesity.

### Clinical correlations of GBS with pregnancy outcomes in late pregnant women

Clinical characteristics of pregnancy outcomes in 380 late pregnant women was recorded, including delivery mode, premature delivery, intrauterine infection, premature rupture of membranes, postpartum hemorrhage, fetal distress, puerperal infection and amniotic fluid contamination (Supplementary Table S1). Correlation analysis suggested that GBS state was positively related to intrauterine infection (*r* = 0.147, *P* = 0.004), premature rupture of membranes (*r* = 0.136, *P* = 0.008), postpartum hemorrhage (*r* = 0.126, *P* = 0.014), fetal distress (*r* = 0.135, *P* = 0.008) and puerperal infection (*r* = 0.135, *P* = 0.008) (Table 2), but not to the mode of delivery, premature delivery or amniotic fluid contamination (Table 2).

**Table 2:** Clinical correlations of GBS with pregnancy outcomes

Pregnancy outcomes	Correlation coefficient	*P*
Caesarean birth	−0.016	0.762
Premature delivery	0.045	0.382
Intrauterine infection	0.147	0.004
Premature rupture of membranes	0.136	0.008
Postpartum hemorrhage	0.126	0.014
Fetal distress	0.135	0.008
Puerperal infection	0.135	0.008
Amniotic fluid contamination	0.010	0.847

### Clinical correlations of GBS with neonatal outcomes

Neonatal outcomes including neonatal infections, neonatal asphyxia, neonatal pneumonia and neonatal sepsis were recorded (Supplementary Table S2). We also analyzed the clinical correlation of GBS with neonatal outcomes. The results showed that GBS status was positively connected with neonatal infections (*r* = 0.148, *P* = 0.004), neonatal pneumonia (*r* = 0.194, *P* < 0.001) and neonatal sepsis (*r* = 0.191, *P* < 0.001) (Table 3). Besides, we also analyzed the effects of delivery mode on neonatal GBS infection, and found a negative association, despite without statistical significance (*r* = −0.016, *P* = 0.762).

**Table 3:** Clinical correlations of GBS with neonatal outcomes

Neonatal outcomes	Correlation coefficient	*P*
Neonatal infections	0.148	0.004
Neonatal asphyxia	0.066	0.201
Neonatal pneumonia	0.194	<0.001
Neonatal sepsis	0.191	<0.001

## Discussion

GBS could be detected in various organs and leads to different diseases [16]. Researches have demonstrated that the overall percentage of GBS carriers is 6.5–36% among late pregnancy women in different regions and races [17–19]. In China, the proportion of pregnant women with GBS was 3.5–32.4% [20]. In the present study, we found that the incidence of GBS colonization in late pregnant women was 12.6%, locating within the range from previous reports. Slight discrepancy might be attributed to the host or environmental conditions or clinical practices involving the management of pregnant women.

In our study, the colonization of GBS was detected using the method of RT-PCR. Currently, there are various detection methods for GBS, including traditional culture, immunochromatographic detection, automatic microbial analysis and identification system and PCR methods [21,22]. The traditional culture method takes a long time, and its operation process is relatively difficult. The detection sensitivity and specificity of immunochromatographic detection are unsatisfactory. Automatic microbial analysis and identification system is a rapid and accurate method for GBS detection, but its application value is limited by high cost. Compared with other available detection methods, PCR method is a rapid, easy and low-cost detection for GBS, and it shows advantages in high sensitivity, specificity and repeatability [22,23]. Thus, PCR was adopted for detection of GBS in our study.

Previous studies found that several pregnancy-related chronic diseases might be associated with maternal GBS colonization [24]. Besides, GBS carriage may be intermittent, the GBS carrier status of pregnant women who with 35–37 weeks gestational age on intrapartum diagnostic sensitivity of GBS carriers was 87% [25]. Moreover, it is benefit to avoid the excessive use or abuse of antibiotics. In our study, we found that positive GBS was more frequent in pregnant women with GDM, PIH and abortion history. Therefore, the findings suggested that pregnant women with the above situations should receive strengthened management and prenatal treatment. In addition, intrapartum antibiotics could be recommended to pregnant women with the above conditions but experiencing no prenatal screening, to prevent poor maternal–infant outcome.

GBS is a leading cause of invasive infections in pregnant women, resulting in neonatal sepsis and meningitis, and associated with a high rates of mortality and morbidity [26,27]. In the present study, we analyzed the relationship of GBS infection with pregnancy and neonatal outcomes. The results indicated that GBS infection was positively associated with pregnancy outcomes, including intrauterine infection, premature rupture of membranes, postpartum hemorrhage, fetal distress and puerperal infection. In addition, we also found that GBS infection was positively associated with neonatal outcomes, such as neonatal infections, neonatal pneumonia and neonatal sepsis. Our results were consistent with those in previous studies. For instance, Shi et al. have reported that maternal GBS at 35–37 weeks gestation can lead to adverse pregnant outcomes due to the increased incidence of intrauterine and neonatal infections [28]. In the study by Mavenyengwa RT et al., GBS colonization was common among pregnant women in Zimbabwe [29]. Besides, we also analyzed the effects of delivery mode on neonatal GBS infection, and found a negative association between them, though it was not statistically significant. Due to the relatively small sample size, further investigations are still required to address the issue.

In conclusion, the late pregnant women with GDM, PIH and abortion history are more likely to be inflected by GBS. Moreover, GBS, infection in late pregnant women may result in adverse effects on maternal and neonatal outcomes. The present study emphasizes the importance of GBS detection among the late pregnant women. Therefore, in clinic, the detection of GBS in pregnancy health care need to be strengthened, especially for the late pregnancy women with high risk factors. The early detection and timely intervention may reduce puerperas and newborn infection, and improve their outcomes.

## Supplementary Material

Supplementary Tables S1-S2

## Abbreviations

GBS: Group B Streptococcus
RT-PCR: real-time polymerase chain-reaction
