# Supplementary material for: The effect of group B streptococcus on maternal and infants’ prognosis in Guizhou, China
Source: Biosci Rep. 2019 Dec 10;39(12):BSR20191575. doi: 10.1042/BSR20191575 (PMC6904771; doi:10.1042/BSR20191575)
Supplement: Supplementary Tables S1-S2 [file BSR-2019-1575_supp.pdf]

**Supplementary Table 1.** Association of GBS colonization with pregnancy outcomes of late pregnant women

| Factors | GBS positive (n=48) | GBS negative (n=332) | $\chi^2$ | *P* |
|---|---|---|---|---|
| Caesarean birth | 21 | 153 | 0.092 | 0.762 |
| Premature delivery | 2 | 7 | 0.768 | 0.381 |
| Intrauterine infection | 10 | 26 | 8.267 | 0.004 |
| Premature rupture of membranes | 14 | 27 | 7.011 | 0.008 |
| Postpartum hemorrhage | 5 | 10 | 6.064 | 0.014 |
| Fetal distress | 20 | 79 | 6.953 | 0.008 |
| Puerperal infection | 8 | 20 | 6.959 | 0.008 |
| Amniotic fluid contamination | 7 | 45 | 0.038 | 0.846 |

**Supplementary Table 2.** Association of GBS colonization with neonatal outcomes

| Factors | GBS positive (n=48) | GBS negative (n=332) | $\chi^2$ | *P* |
|---|---|---|---|---|
| Neonatal infections | 13 | 39 | 8.351 | 0.004 |
| Neonatal asphyxia | 2 | 5 | 1.642 | 0.200 |
| Neonatal pneumonia | 3 | 1 | 14.249 | 0.000 |
| Neonatal sepsis | 2 | 0 | 13.907 | 0.000 |
